# Etoposide-mediated interleukin-8 secretion from bone marrow stromal cells induces hematopoietic stem cell mobilization

**DOI:** 10.1186/s12885-020-07102-x

**Published:** 2020-07-02

**Authors:** Ka-Won Kang, Seung-Jin Lee, Ji Hye Kim, Byung-Hyun Lee, Seok Jin Kim, Yong Park, Byung Soo Kim

**Affiliations:** 1 Division of Hematology-Oncology, Department of Internal Medicine, Korea University School of Medicine, 73, Goryeodae-ro, Seongbuk-gu, Seoul, 02841 South Korea; 2 Institute of Stem Cell Research, Korea University, Seoul, South Korea; 3 Department of Biomedical and Science, Graduate School of Medicine, Korea University, Seoul, South Korea; 4 Division of Hematology-Oncology, Department of Internal Medicine, Sungkyunkwan University School of Medicine, Seoul, South Korea

**Keywords:** Hematopoietic stem cell mobilization, Etoposide, Cyclophosphamide, G-CSF

## Abstract

**Background:**

We assessed the mechanism of hematopoietic stem cell (HSC) mobilization using etoposide with granulocyte-colony stimulating factor (G-CSF), and determined how this mechanism differs from that induced by cyclophosphamide with G-CSF or G-CSF alone.

**Methods:**

We compared the clinical features of 173 non-Hodgkin’s lymphoma patients who underwent autologous peripheral blood stem cell transplantation (auto-PBSCT). Additionally, we performed in vitro experiments to assess the changes in human bone marrow stromal cells (hBMSCs), which support the HSCs in the bone marrow (BM) niche, following cyclophosphamide or etoposide exposure. We also performed animal studies under standardized conditions to ensure the following: exclude confounding factors, mimic the conditions in clinical practice, and identify the changes in the BM niche caused by etoposide-induced chemo-mobilization or other mobilization methods.

**Results:**

Retrospective analysis of the clinical data revealed that the etoposide with G-CSF mobilization group showed the highest yield of CD34+ cells and the lowest change in white blood cell counts during mobilization. In in vitro experiments, etoposide triggered interleukin (IL)-8 secretion from the BMSCs and caused long-term BMSC toxicity. To investigate the manner in which the hBMSC-released IL-8 affects hHSCs in the BM niche, we cultured hHSCs with or without IL-8, and found that the number of total, CD34+, and CD34+/CD45- cells in IL-8-treated cells was significantly higher than the respective number in hHSCs cultured without IL-8 (*p* = 0.014, 0.020, and 0.039, respectively). Additionally, the relative expression of *CXCR2* (an IL-8 receptor), and *mTOR* and *c-MYC* (components of IL-8-related signaling pathways) increased 1 h after IL-8 treatment. In animal studies, the etoposide with G-CSF mobilization group presented higher IL-8-related cytokine and MMP9 expression and lower SDF-1 expression in the BM, compared to the groups not treated with etoposide.

**Conclusion:**

Collectively, the unique mechanism of etoposide with G-CSF-induced mobilization is associated with IL-8 secretion from the BMSCs, which is responsible for the enhanced proliferation and mobilization of HSCs in the bone marrow; this was not observed with mobilization using cyclophosphamide with G-CSF or G-CSF alone. However, the long-term toxicity of etoposide toward BMSCs emphasizes the need for the development of more efficient and safe chemo-mobilization strategies.

## Background

Successful autologous peripheral blood stem cell transplantation (auto-PBSCT) for hematological malignancies requires harvesting a sufficient number of human hematopoietic stem cells (hHSCs) mobilized from the bone marrow (BM) to the peripheral blood (PB). In clinical practice, the mobilization protocols generally include chemotherapy and granulocyte colony-stimulating factor (G-CSF) (chemo-mobilization), as restricting the cancer burden during mobilization is crucial. Since the first clinical application of G-CSF by Dührsen et al. in 1988 [1], cyclophosphamide chemo-mobilization has been commonly used for chemo-mobilization [2, 3]. Cyclophosphamide induces the release of stress signals that cause inflammation, thereby activating the host immune system, which may increase hHSC mobilization [4, 5]. However, this protocol has some disadvantages, including—primarily—the unpredictability of the number of hHSCs that can be collected from the PB and the possibility of mobilization-related toxicities, such as febrile neutropenia [5–7]. Reiser et al. had first reported the use of etoposide as an alternative to cyclophosphamide to effectively mobilize PBSCs in patients in whom cyclophosphamide-induced chemo-mobilization had failed [8]; this led to studies on etoposide-induced chemo-mobilization (Supplementary Material 1: Table S1) [9–13]. However, concerns regarding the use of etoposide include its inhibition of topoisomerase 2, which damages DNA. Cancer patients undergoing chemotherapy regimens that include etoposide, have been reported to experience secondary hematological malignancies [14, 15]. Moreover, Gibson et al. demonstrated that etoposide could damage human bone marrow stromal cells (hBMSCs) [16]. These findings suggest that etoposide may influence the BM niche by not only enhancing hHSC mobilization but also by inducing BM damage. Therefore, the mechanism underlying etoposide-induced mobilization may differ from that of G-CSF- or cyclophosphamide-induced mobilization, which proceeds through the demargination of HSCs from the BM to PB due to systemic inflammation [17]. However, to date, this topic appears to have received little attention. Furthermore, verification of the mobilization mechanisms may be difficult due to the interference of complex physical conditions in patients, which could confound the interpretation of the associated clinical findings. To overcome these barriers, we designed a three-step study involving the following: 1) analysis of clinical data associated with auto-PBSCT in patients with non-Hodgkin’s lymphoma (NHL); 2) in vitro experiments to assess the changes in hBMSCs, which support HSCs in the BM niche, after exposure to cyclophosphamide or etoposide; and 3) in vivo animal studies under standardized conditions to exclude confounding factors, mimic conditions of clinical practice, and identify changes in the BM niche caused by etoposide-induced chemo-mobilization or other mobilization protocols.

## Methods

### Clinical data

The clinical data of patients with Non-Hodgkin Lymphoma (NHL) who underwent PB stem cell collection (PBSCC) at the Korea University Anam Hospital and the Samsung Medical Center, from 2005 to 2019, was retrospectively analyzed, and a retrospective chart review was conducted. Both these studies were approved by an internal board of the Korea University Anam Hospital (IRB No. 2019AN0386) and the Samsung Medical Center (2019–09–085-001).

### Primary hBMSC culture

The internal review board of the Korea University Anam Hospital (IRB No. 2015AN0267) approved all the procedures. Written informed consent was obtained from all subjects. The subjects were healthy individuals who donated BM via BM harvesting. A total of 20 mL BM was collected from each subject. Mononuclear cells (MNCs) were separated using Ficoll-Paque™ Plus medium (GE Healthcare Life Sciences, Seoul, South Korea); the remaining cells were cultured in mesenchymal stem cell growth medium (Lonza, Walkersville, MD, USA). In this study, we used isolated hBMSCs within five passages from the start of the subculture and routinely tested to confirm the absence of mycoplasma by the e-Myco™ VALiD mycoplasma PCR detection kit (iNtRON, Burlington, MA, USA).

### Flow cytometry

Antibodies against anti-human CD73-PE, CD90-PE, CD105-PE, CD34-FITC, and CD45-PE (Becton Dickinson, San Jose, CA, USA) were used at 1:100 dilution. Cells were analyzed using FACSCalibur™ (Becton Dickinson).

### Chemotherapeutic agents and cytotoxicity assay

Commercially available preparations of cyclophosphamide (Endoxan injection, 500 mg; Boxter Inc., Seoul, South Korea) and etoposide (Lastet injection, 100 mg/5 mL; Dong-A Inc., Seoul, South Korea) were used. Cell Counting Kit-8 (CCK-8 assay, Dojindo Laboratories, Japan) was used for the cytotoxicity assays, according to the manufacturer’s instructions. Absorbance was measured at 450 nm using a SpectraMax Plus 384 spectrophotometer (Molecular Devices Corporation, CA, USA).

### Human and mouse cytokine arrays

The Human Cytokine Antibody Array C1000 and Mouse Cytokine Antibody Array C1000 (both from Ray Biotech, GA, USA) were used, according to the manufacturer’s instructions. Images were acquired using a ChemiDoc™ Touch Imaging System (Bio-Rad, Hercules, CA, USA) and quantified using ImageJ (National Institutes of Health, MD, USA). Signal was normalized using the internal positive controls and the background with the RayBio® Antibody Array Analysis Tool (Ray Biotech).

### Apoptosis and cell cycle analysis

Apoptosis analysis was performed using the EzWay Annexin V-FITC Apoptosis Detection Kit (Koma Biotech Inc., Seoul, South Korea). Cell-cycle distribution analysis was performed using propidium iodide at 50 mg/mL (Sigma-Aldrich, catalog no. P4170). Both assays were performed according to the manufacturers’ instructions.

### HSC culture and IL-8 treatment

Human BM CD34+ HSCs were purchased from Lonza (catalog no. 2 M-101) and cultured in Stemline® II Hematopoietic Stem Cell Expansion Medium (Sigma-Aldrich, catalog no. S0192) containing 100 ng/mL stem cell factor, thrombopoietin, and G-CSF (all obtained from R&D Systems, Inc., Minneapolis, MN, USA). Recombinant human IL-8/CXCL8 protein was acquired from R&D Systems (catalog no. 208-IL).

### Quantitative reverse transcription-polymerase chain reaction (qRT-PCR)

Total RNA was isolated from cells using the Qiagen RNeasy kit (Qiagen, Hilden, Germany) and quantified using a NanoDrop spectrophotometer (Thermo Fisher Scientific, Inc., Waltham, MA, USA). cDNA was synthesized using 2 μg total RNA as a template in a 20-μL reaction mixture containing oligos, primers, and Superscript II reverse transcriptase (Thermo Fisher Scientific, Inc.), according to the manufacturer’s instructions. Synthesized cDNA was amplified using the iQ SYBR Green qPCR Master Mix (Bio-Rad) on a Bio-Rad iCycler iQ (Bio-Rad). Comparative threshold cycle values were normalized to those of glyceraldehyde-3-phosphate dehydrogenase. The primers used are described in Supplementary Material 2: Table S2. To compare the difference in mRNA expression, relative quantification was performed using the delta-delta Ct method [18]. In brief, the ΔCt value was obtained after normalization based on the internal control (GAPDH), and the ΔΔCt value was obtained based on the control group. We then used 2^-ΔΔCt^ to calculate the fold change.

### Mice

All experimental procedures using animals complied with the guidelines of the Laboratory Animal Research Center of the Korea University College of Medicine (IRB No. KOREA-2017-0176). A total of 87 C57BL/6 N mice were purchased from Orient Bio (Seongnam, South Korea). Mice, 8 weeks of age and with a body weight of 20 g, were maintained in polypropylene cages under specific pathogen-free conditions, with light/dark 12-h cycles, at 21 ± 2 °C, and had ad libitum access to a maintenance diet. Sample sizes were calculated using a pilot study and the G*Power program (http://www.gpower.hhu.de/). All analyses were conducted blindly to minimize the effects of subjective bias.

### Protocol for HSC mobilization in mice

The mouse model of HSC mobilization was designed based on protocol used in human patients (Fig. 4a–b). A previously reported model of cyclophosphamide chemo-mobilization was used in this study [19]. Due to the apparent lack of a related animal model, we developed a new model of etoposide chemo-mobilization. Mice were injected intraperitoneally with 4 mg cyclophosphamide (≈ 200 mg/kg) on day 1 (D1) or with 0.8 mg etoposide (≈ 40 mg/kg) on days 1 and 2 (D1, D2). Subsequently, 5 μg human G-CSF (250 μg/kg per day; Leucostim prefilled syringe INJ, Dong-A Inc.) was administered daily as a single subcutaneous injection, on each successive day from day 3, for a total of 4 days. All mice were euthanized on D7 by cardiac puncture and cervical dislocation under anesthesia. On day 7 (D7), we isolated hematopoietic progenitor cells (HPCs) using an EasySep™ Mouse Hematopoietic Progenitor Cell Isolation Kit and performed colony-forming unit (CFU) assays using MethoCult™ GF M3434 medium (both from Stem Cell Technologies, Vancouver, BC, Canada), according to the manufacturer’s instructions.

### Enzyme-linked immunosorbent assay (ELISA)

Plasma levels of stromal cell-derived factor-1 (SDF-1), matrix metalloproteinase-2 (MMP2), and matrix metalloprotease-9 (MMP9) in mice were measured using the Magnetic Luminex® Screening Assay (R&D Systems), according to the manufacturer’s instructions.

### Immunohistochemistry of BM sections

Immunohistochemistry (IHC) was performed on 3-μm formalin-fixed, paraffin-embedded sections from the BM. The following primary antibodies were used: anti- keratinocyte-derived cytokine (KC) (Cloud-Clone Corp., Houston, TX, USA; catalog no. PAA041Mu01; 1:50), anti-macrophage inflammatory protein 2 (MIP-2) (Cloud-Clone Corp.; catalog no. PAB603Mu01; 1:100), anti-lipopolysaccharide-inducible CXC chemokine (LIX) (Cloud-Clone Corp.; catalog no. PAA860Mu01; 1:100), anti-MMP2 (Abcam; catalog no. ab37150; 1:200), anti-MMP9 (Abcam; catalog no. ab38898; 1:200), and anti-SDF-1 (Abcam; catalog no. ab9797; 1:500). In the case of anti-KC, anti-MIP-2, anti-LIX, anti-MMP9, and anti-SDF-1, antigen retrieval was performed using a citrate buffer. All slides were scanned using a virtual microscopy scanner (Axio Scan Z1 scanner; Carl Zeiss, Jena, Germany); positive contributions were calculated by summing the highly positive, positive, and low-positive fractions for each staining using the IHC profiler Plugin of ImageJ [20].

### Statistical analysis

Patient demographics and baseline characteristics were compared using Kruskal–Wallis H and Chi-square tests. Multivariate analysis using the Cox proportional hazards method was performed. Mann–Whitney U, Student’s *t*-tests, and analysis of variance were used to analyze differences in data from the in vitro and in vivo experiments, based on the variables involved. A post hoc analysis with Bonferroni correction was performed when statistical differences were identified among the three groups. Data analysis was performed using IBM SPSS Statistics for Windows, version 25.0 (IBM Corp., NY, USA). Significant differences are denoted by *p*-values < 0.05.

## Results

### Etoposide-induced chemo-mobilization is highly effective and exhibits different clinical features, compared to the other mobilization methods

**Fig. 1:**
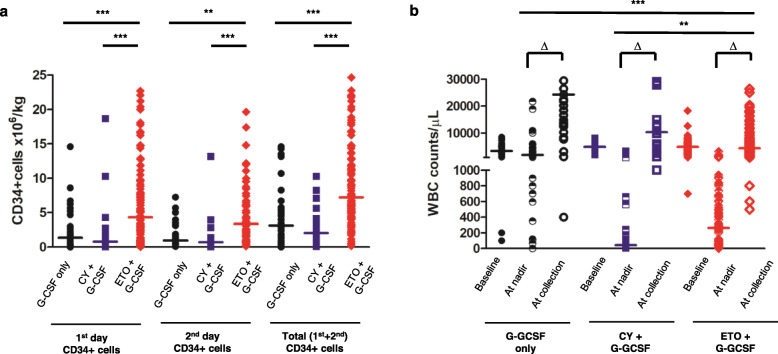
Yield of CD34+ cells and changes in white blood cell counts based on the mobilization method. **a** Data from 173 patients diagnosed with lymphoma who underwent peripheral blood stem cell collection (G-CSF only, *n* = 33; cyclophosphamide + G-CSF, *n* = 24; etoposide + G-CSF, *n* = 116) were analyzed. The highest yield of CD34+ cells was observed for etoposide + G-CSF [(1st day: G-CSF only: 1.36 (0.01–14.60); cyclophosphamide + G-CSF, 0.81 (0.05–18.70); etoposide + G-CSF, 4.32 (0.03–32.77), 2nd day: G-CSF-only, 0.96 (0.09–7.25); cyclophosphamide + G-CSF, 0.70 (0.06–13.20); etoposide + G-CSF, 3.37 (0.14–32.60), Total: G-CSF only, 3.13 (0.01–14.60); cyclophosphamide + G-CSF, 2.05 (0.12–31.9); etoposide + G-CSF, 7.22 (0.18–59.20)]. **b** The change in white blood cell (WBC) counts at the nadir and at the time of collection during mobilization was the lowest for the etoposide + G-CSF group among the three groups (∆WBC: G-CSF only, 15,305 (− 1412–574,000); cyclophosphamide + G-CSF, 10,320 (916–70,884); etoposide + G-CSF, 3770 (254–120,780)). Note: ‘At the nadir’ refers to the lowest WBC value during chemotherapy before peripheral blood stem cell collection. ‘∆WBC’ refers to the increase in WBC counts from the nadir to the time of peripheral blood stem cell collection. Note: *** *p* < 0.001 after Bonferroni correction; ** *p* < 0.01 after Bonferroni correction. Note: Values are reported as the median with range. Abbreviations: G-CSF, granulocyte colony-stimulating factor; CY, cyclophosphamide; ETO, etoposide

We analyzed data from 173 patients with NHL who underwent PBSCC in the presence of the following chemotherapeutic agents: G-CSF only, *n* = 33; cyclophosphamide + G-CSF, *n* = 24; and etoposide + G-CSF, *n* = 116. The baseline characteristics of the patients are summarized in Table 1. The highest yield of CD34+ cells was observed for etoposide + G-CSF (Fig. 1a), a result that remained significant even after adjusting for baseline characteristics (Supplementary Material 3: Table S3). The increase in white blood cell (WBC) count (from the nadir to the time of PBSCC) was modest for etoposide + G-CSF, compared with that for G-CSF only and cyclophosphamide + G-CSF (Fig. 1b). In etoposide + G-CSF, WBC counts at the nadir (cyclophosphamide + G-CSF, 41 (9–3258); etoposide + G-CSF, 262 (1–3160)) were higher, and those at the time of PBSCC (cyclophosphamide + G-CSF, 10,350 (1000–70,900); etoposide + G-CSF, 4380 (500–122,150)) were lower than the WBC counts in cyclophosphamide + G-CSF (*p* = 0.056 and 0.005, respectively). Previous studies have reported a positive correlation between the degree of WBC count increase during mobilization and the increase in CD34+ cell yield [21–23]. In the present study, etoposide-induced chemo-mobilization led to the highest CD34+ cell yield, despite the fact that the differences in WBC counts between the nadir and the time of PBSCC were the lowest. Therefore, we suspected that the mechanism underlying HSC mobilization by etoposide might differ from that of G-CSF only and cyclophosphamide. However, our hypothesis must be confirmed because there was heterogeneity among patients in each group and because of the presence of other confounding factors.

**Table 1:** Baseline characteristics

Baseline characteristics	G-CSF only (*n*= 33)	CY+G-CSF (*n*= 24)	ETO+G-CSF (*n*= 116)	*p*-value
**Median age (in years) (range)**	43.0 (17.0–67.0)	46.5 (20.0–62.0)	52 (21.0–65.0)	**0.003**
**Male:female ratio**	2.00	2.43	1.23	0.243
**Histology,*n*(%)**				0.139
**Hodgkin lymphoma**	3 (9.1)	4 (16.7)	5 (4.3)	
**Non-Hodgkin lymphoma**				
**B cell**	15 (45.5)	14 (58.3)	65 (56.0)	
**T cell**	15 (45.5)	6 (25.0)	46 (39.7)	
**Disease stage,*n*(%)**				0.342
**Limited stage (Stage I-II)**	8 (24.2)	3 (12.5)	31 (26.7)	
**Advanced stage (Stage III-IV)**	25 (75.8)	21 (87.5)	85 (73.3)	
**Bone marrow involvement at dagnosis,*n*(%)**	5 (15.2)	6 (25.0)	30 (27.5)	0.472
**Number of previous chemotherapy treatments (range)**	2 (1-3)	2 (1–3)	1 (1-5)	0.139
**Disease status before mobilization,*n*(%)**				0.080
**Complete remission**	12 (36.4)	10 (41.7)	52 (44.8)	
**Partial remission**	19 (57.6)	12 (50.0)	47 (40.5)	
**Dose, total (mg/m**^**2**^**) (range)**	━	3,000 (1,000–3,000)	750 (375–750)	━
**Time from diagnosis to start of mobilization (months) (range)**	7.6 (3.7–63.3)	16.6 (0.7-59.9)	6.2 (1.5–148.0)	**0.003**
**Median follow-up duration after mobilization (months) (range)**	12.2 (0.1–96.0)	37.8 (0.1–92.6)	13.0 (1.4–76.8)	━
**Transplantation done,*n*(%)**	29 (90.6)	16 (72.7)	112 (96.6)	━

### Etoposide increases IL-8 secretion from BMSCs and causes long-term MBSC toxicity

**Fig. 2:**
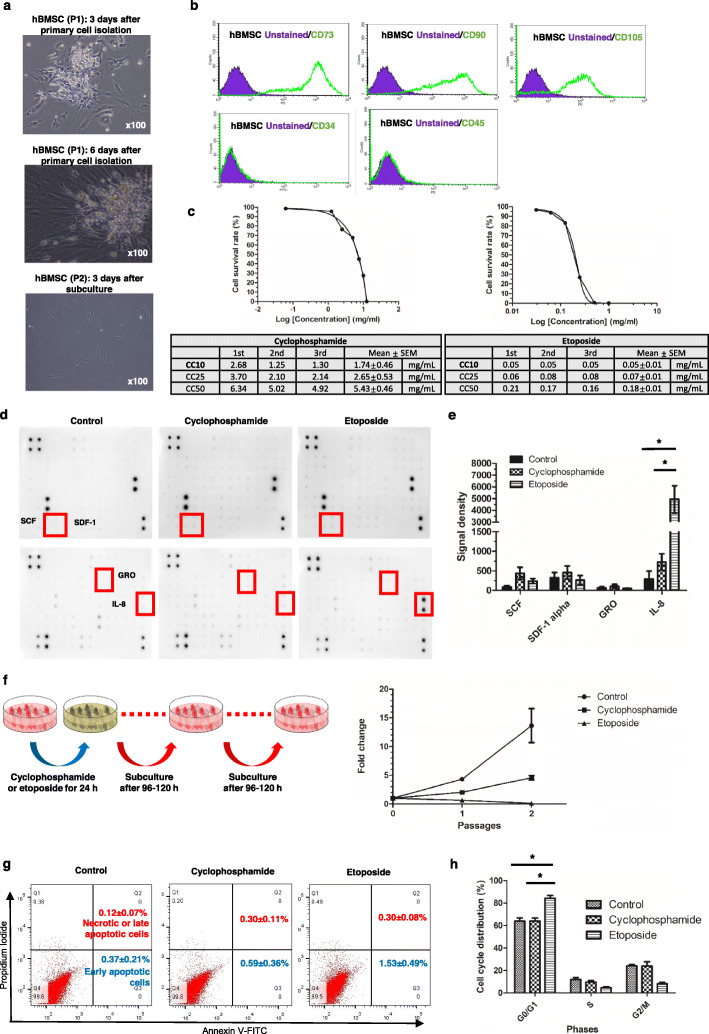
Primary culture of human bone marrow stromal cells and results of the cytotoxicity assays, cytokine arrays, and apoptosis and cell cycle analyses. **a** Mononuclear cells were collected from a healthy donor during bone marrow harvest. After 1–2 weeks of primary culture, adherent cells showed spindle-shaped morphology and reached 65–70% confluence. **b** Flow cytometry indicated that these cells were positive for the human bone marrow stromal cell (hBMSC) markers CD73, CD90, and CD105 and negative for the hematopoietic stem cell markers CD34 and CD45. These results indicate that hBMSCs were properly isolated. **c** Cytotoxic concentration (CC) 10, CC 25, and CC 50, defined as the concentrations sufficient to cause the death of 10, 25, and 50% of viable hBMSCs, were calculated for various concentrations of cyclophosphamide and etoposide. **d** hBMSCs were cultured in normal saline (control group, *n* = 4), cyclophosphamide (dose of CC10, *n* = 5), or etoposide (dose of CC10, *n* = 5) for 24 h. Human cytokine analysis was performed with the conditioned media. The level of IL-8, a mobilization-associated cytokine, was significantly higher in the etoposide-treated group than that in the cyclophosphamide-treated group (*p* = 0.021 after Bonferroni correction). **f** Expansion of etoposide-treated hBMSCs was significantly lower than that of cyclophosphamide-treated hBMSCs in both P1 and P2 (control, *n* = 7; cyclophosphamide, *n* = 7; etoposide, *n* = 7; both, *p* < 0.001 after Bonferroni correction). **g** No differences in the numbers of early apoptotic and necrotic cells or late apoptotic cells were observed among the groups (control, *n* = 4; cyclophosphamide, *n* = 7; etoposide, *n* = 7). As a negative control, hBMSCs treated only with normal saline were used. The values within the figures represent the mean ± standard error in repeated experiments. All experimental data of representative figures are presented as Supplementary Material 6: Fig. S3. **h** Etoposide-treated hBMSCs showed a higher proportion of cells arrested in the G0/G1 phase of the cell-cycle than the cyclophosphamide-treated and untreated hBMSCs (control, *n* = 3; cyclophosphamide, *n* = 3; etoposide, *n* = 3; *p* = 0.03 and *p* = 0.01 after Bonferroni correction, respectively). Note: * *p* < 0.05 after Bonferroni correction. Note: Values are reported as the mean ± standard error of the mean (SEM). Abbreviations: P1, passage 1; P2, passage 2; CC, cytotoxic concentration

hBMSCs, which constitute the major cell component of the BM niche [24], were isolated from BM (Fig. 2a–b) and treated with various concentrations of cyclophosphamide (0–12.5 mg/mL) or etoposide (0–2.0 mg/mL) for 24 h. Drug concentrations sufficient to cause the death of 10, 25, and 50% of the viable hBMSCs were defined as cytotoxic concentration (CC) 10, CC 25, and CC 50, respectively (Fig. 2c). Data regarding the blood concentrations of the two drugs from patients receiving high-dose cyclophosphamide or etoposide treatment was compiled from the literature. For high-dose cyclophosphamide treatment (1850–7000 mg/m^2^), the maximum reported serum concentration (C_max_) was 2.664 mg/mL [25, 26]. For high-dose etoposide treatment (1480–1665 mg/m^2^), the reported C_max_ was 0.1 mg/mL [27, 28]. Based on this information, the CC10 was selected as the drug concentration for further experiments.

hBMSCs were cultured in a medium containing normal saline (control group, *n* = 4), cyclophosphamide (dose of CC10, *n* = 5), or etoposide (dose of CC10, *n* = 5) for 24 h; subsequently, human cytokine analysis was performed using the conditioned media. The level of IL-8, a mobilization-associated cytokine [29, 30], was significantly higher in the etoposide-treated group than in the cyclophosphamide-treated group (*p* = 0.021 after Bonferroni correction) (Fig. 2d–e). Other mobilization-associated cytokines showed no significant differences among the groups.

The degree of expansion of etoposide-treated hBMSCs was significantly lower than that of cyclophosphamide-treated hBMSCs for all passages (*p* < 0.001 after Bonferroni correction for both) (Fig. 2f). No significant differences in apoptosis were observed among the groups (Fig. 2g). However, cell-cycle analysis revealed a significantly higher proportion of etoposide-treated hBMSCs arrested in the G0/G1 phase than cyclophosphamide-treated and untreated hBMSCs (*p* = 0.03 and *p* = 0.01 after Bonferroni correction, respectively; Fig. 2h).

### IL-8 enhances HSC expansion and is associated with CXCR2, mTOR, and c-*MYC* activation

**Fig. 3:**
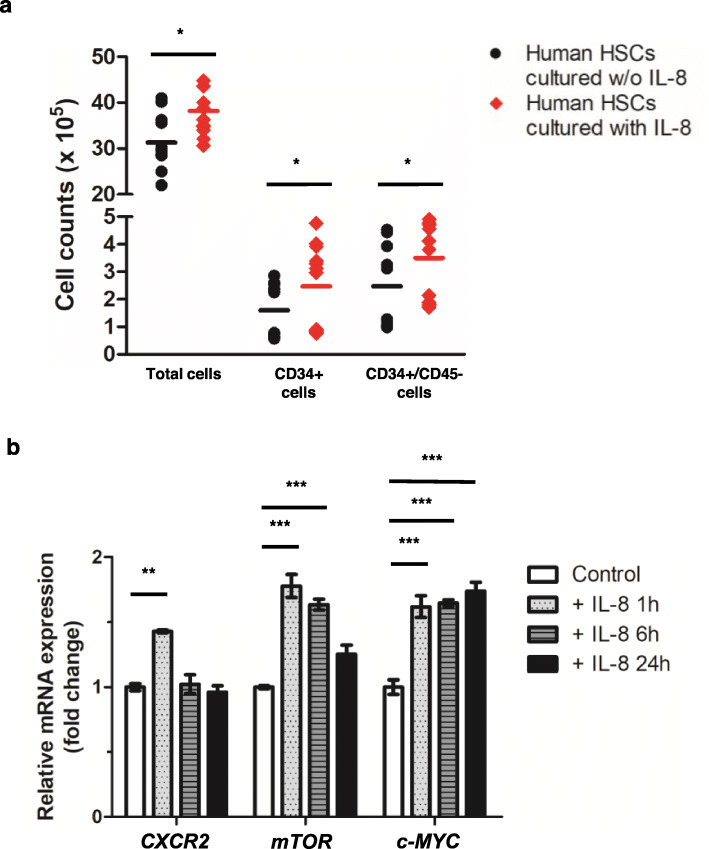
Effects of increased IL-8 levels on hematopoietic stem cells. **a** Conditioned media was collected from 24-h cultures of healthy hBMSCs grown in mesenchymal stem cell growth medium. Subsequently, 2.5 × 10^6^ hHSCs were cultured for 24 h in conditioned media in the presence *n* = 12) and absence (*n* = 12) of IL-8 (100 ng/mL). The numbers of total, CD34+, and CD34+/CD45- cells were significantly higher in the hHSCs cultured in the presence of IL-8, compared to those in cells cultured without IL-8 (*p* = 0.014, *p* = 0.020, and *p* = 0.039, respectively). **b** The relative expression of *CXCR2*, *mTOR*, and c-*MYC* increased at 1 h after IL-8 treatment. The expression of *CXCR2* returned to normal after 6 h of IL-8 treatment, and the expression of mTOR gradually decreased at 6 and 24 h after IL-8 treatment. In the case of c*-MYC*, the increased expression lasted up to 24 h. Each experiment was repeated thrice. Note: *** *p* < 0.001; ** *p* < 0.01; * *p* < 0.05. Note: Values are reported as the median with range (A) and the mean ± SEM (B). Abbreviations: hBMSCs, human bone marrow stromal cells; hHSC, human hematopoietic stem cell

We observed significantly increased IL-8 secretion from hBMSCs treated with etoposide, compared to that from hBMSCs treated with cyclophosphamide. To investigate the manner in which the hBMSC-released IL-8 affects hHSCs in the BM niche, we cultured 2.5 × 10^6^ hHSCs with 100 ng/mL IL-8 (*n* = 12) or without IL-8 (*n* = 12) for 24 h in a conditioned medium collected from 24-h cultures of healthy hBMSCs grown in mesenchymal stem-cell growth medium. Previous experiments had determined the distribution of human cytokines in this conditioned medium (Fig. 2d, control group) and had identified the relatively low IL-8 expression in this medium (Fig. 2e, control group). The numbers of total, CD34+, and CD34+/CD45- cells determined using a hemocytometer and flow cytometric analysis of CD34+ cells cultured with IL-8 were significantly higher than those of cells cultured without IL-8 (*p* = 0.014, 0.020, and 0.039, respectively) (Fig. 3a). To identify the mechanism underlying the effect of IL-8 on hHSCs, the expression of *CXCR2* (an IL-8 receptor) and *mTOR* and c-*MYC* (components of IL-8-related signaling pathways) was measured by qRT-PCR. The relative expression of *CXCR2*, *mTOR*, and c-*MYC* increased at 1 h after IL-8 treatment (Fig. 3b). The expression of *CXCR2* returned to normal 6 h after IL-8 treatment, and the expression of mTOR gradually decreased at 6 and 24 h after IL-8 treatment. In the case of c-*MYC*, the increased expression lasted up to 24 h.

### Etoposide-induced chemo-mobilization increases IL-8-associated cytokine levels, especially in the BM

**Fig. 4:**
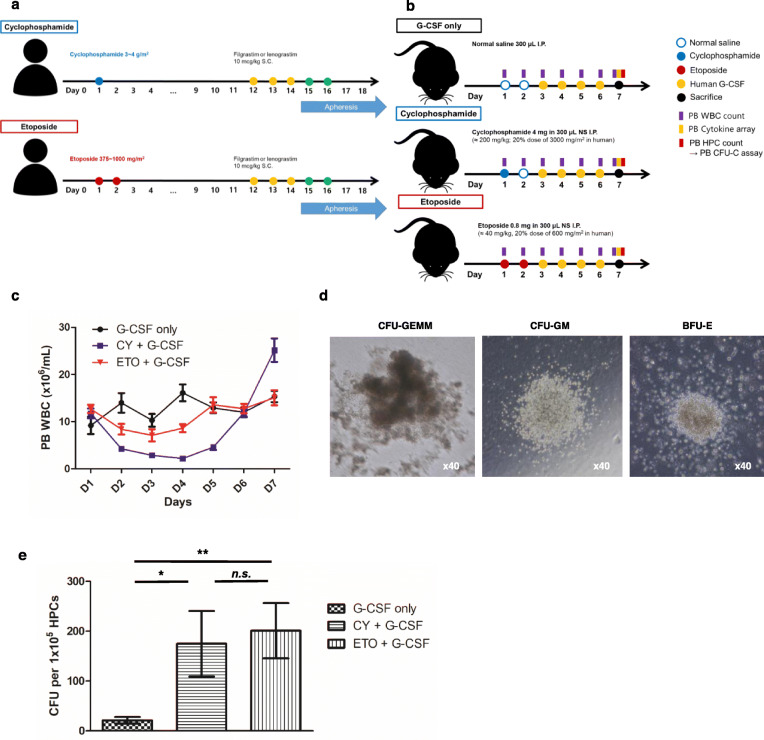
Mouse model of peripheral blood hematopoietic stem cell mobilization. **a**, **b**, **c** The mouse model of hematopoietic stem cell (HSC) mobilization was designed based on a protocol used in human patients (G-CSF only, *n* = 8; cyclophosphamide + G-CSF, *n* = 8; etoposide + G-CSF, *n* = 8). **d** On day 7 (D7) of the protocol, HPCs were isolated from the peripheral blood and CFUs (CFU-granulocytes, erythrocytes, monocytes, and megakaryocytes; CFU-granulocytes, macrophages; and burst-forming unit-erythroid) were counted. The presented pictures were obtained in the control group (G-CSF only). **e** The cyclophosphamide-treated (total 200 mg/kg) and etoposide-treated (total 80 mg/kg) groups showed a higher number of CFUs than the G-CSF only group (*p* = 0.021 and 0.003 after Bonferroni correction, respectively). No significant difference was observed in the total number of CFUs between the cyclophosphamide-treated (200 mg/kg) and etoposide-treated (80 mg/kg) groups (G-CSF only, *n* = 5; cyclophosphamide + G-CSF, *n* = 5; etoposide + G-CSF, *n* = 5). Note: ** *p* < 0.01 after Bonferroni correction; * *p* < 0.05 after Bonferroni correction. Note: Values are reported as the mean ± SEM. Abbreviations: S.C., subcutaneous injection; I.P., intraperitoneal injection; NS, normal saline; G-CSF, granulocyte colony-stimulating factor; CY, cyclophosphamide; ETO, etoposide; CFU, colony-forming unit; GEMM, granulocytes, erythrocytes, monocytes, and megakaryocytes; GM, granulocytes, macrophages; BFU-E, burst forming unit-erythroid; n.s., not significant

We developed mouse models for PB HSC mobilization based on the actual mobilization protocol used in human patients (G-CSF only, *n* = 8; cyclophosphamide + G-CSF, *n* = 8; etoposide + G-CSF, *n* = 8; Fig. 4a–b). Changes in WBC counts at the nadir and at the time of collection (D7) showed patterns similar to those observed in clinical settings (Figs. 1b and 4c). On D7, HPCs were isolated from the PB, and CFUs (CFU-granulocytes, erythrocytes, monocytes, and megakaryocytes; CFU-granulocytes, macrophages; and burst forming unit-erythroids) were counted (Fig. 4d). The cyclophosphamide-treated (total 200 mg/kg) and etoposide-treated (total 80 mg/kg) groups showed a higher number of CFUs than the G-CSF only group (*p* = 0.021 and 0.003 after Bonferroni correction, respectively). No significant differences in the total number of CFUs were observed between the cyclophosphamide-treated (total 200 mg/kg) and etoposide-treated (total 80 mg/kg) groups (G-CSF only, *n* = 5; cyclophosphamide + G-CSF, *n* = 5; etoposide + G-CSF, *n* = 5; Fig. 4e). Thus, this condition might be appropriate to investigate the differences in the mechanisms underlying etoposide-induced and other compound-induced chemo-mobilization.

**Fig. 5:**
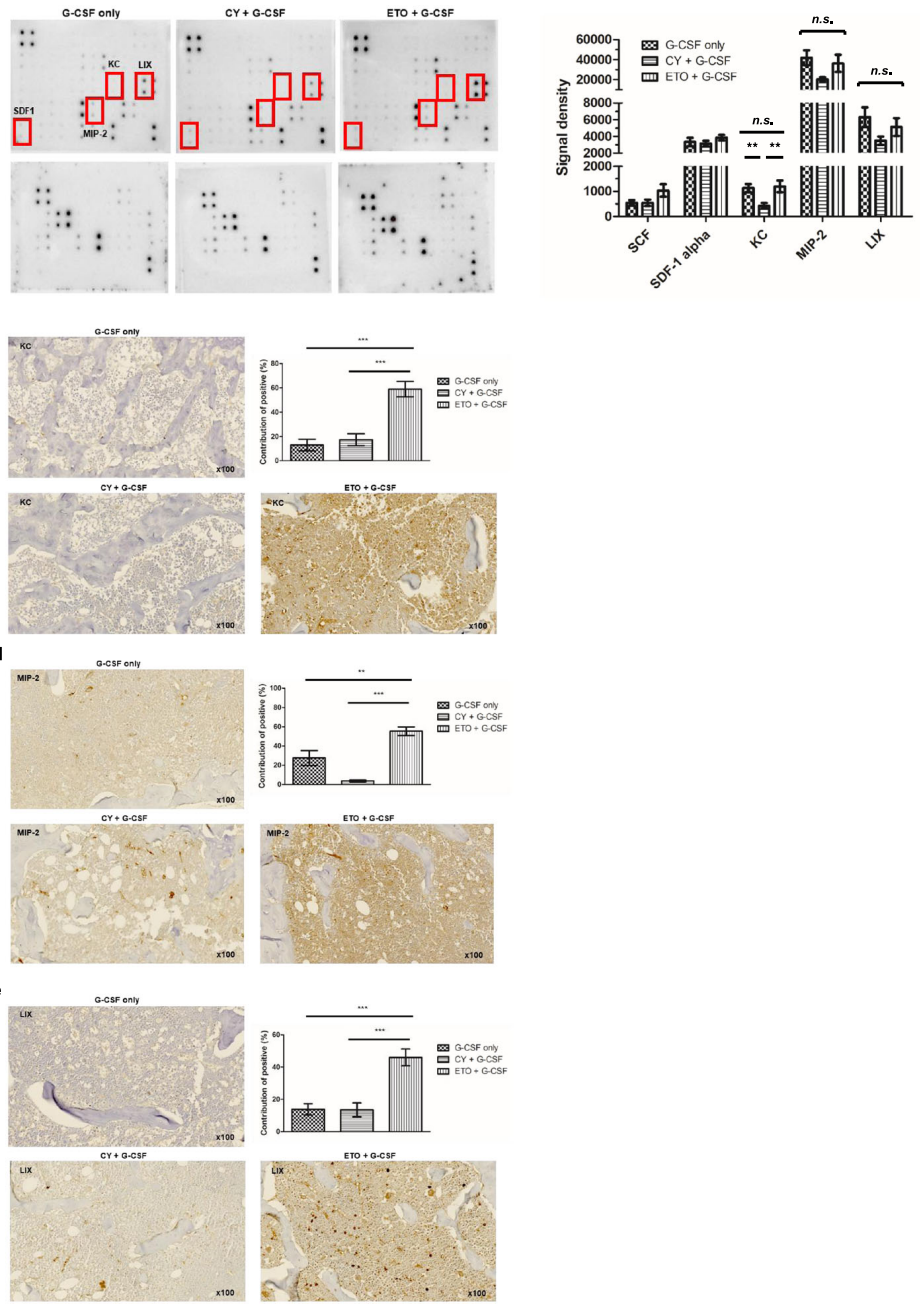
Keratinocyte-derived cytokine (KC), macrophage inflammatory protein 2 (MIP-2), and lipopolysaccharide-inducible CXC (LIX) expression in the mouse model of peripheral blood hematopoietic stem cell mobilization. **a**, **b** Plasma cytokine analysis was performed in the mouse model on day 7. Levels of KC, MIP-2, and LIX (IL-8 homologs in mice) were measured (G-CSF only, *n* = 9; cyclophosphamide + G-CSF, *n* = 9; etoposide + G-CSF, *n* = 9). KC levels significantly increased in the etoposide-treated group, compared with those in the cyclophosphamide-treated group (*p* = 0.001 after Bonferroni correction). Levels of the other IL-8 homologs, MIP-2 and LIX, were also increased in the etoposide-treated group but did not show significant differences compared to the cyclophosphamide-treated group. **c**, **d**, **e** To confirm local changes in KC, MIP-2, and LIX in the bone marrow, we quantified IHC images using the IHC profiler plugin of the ImageJ. KC increased significantly in the etoposide-treated group, compared to that in the G-CSF-only and cyclophosphamide-treated groups (*p* < 0.001 and *p* < 0.001 after Bonferroni correction, respectively). Levels of the other IL-8 homologs, MIP-2 and LIX, increased significantly in the etoposide-treated group, compared to those in the G-CSF-only group and cyclophosphamide-treated group (MIP-2, *p* = 0.004 and *p* < 0.001 after Bonferroni correction, respectively; LIX, *p* < 0.001 and *p* < 0.001 after Bonferroni correction, respectively). Note: *** *p* < 0.001 after Bonferroni correction; ** *p* < 0.01 after Bonferroni correction. Note: Values are reported as the mean ± SEM. Abbreviations: G-CSF, granulocyte colony-stimulating factor; CY, cyclophosphamide; ETO, etoposide; n.s., not significant; IHC, immunohistochemistry

Plasma cytokine levels in whole blood collected from mice on D7 were analyzed. The levels of KC, MIP-2, and LIX, which are IL-8 homologs in mice [31–33], were measured (G-CSF only, *n* = 9; cyclophosphamide + G-CSF, *n* = 9; etoposide + G-CSF, *n* = 9). The level of KC was significantly increased in the etoposide-treated group, compared with that in the cyclophosphamide-treated group (*p* = 0.001 after Bonferroni correction). The levels of the other IL-8 homologs, MIP-2 and LIX, were also increased in the etoposide-treated group, compared with those in the cyclophosphamide-treated group; however, the differences were not significant. None of the three homologs showed significant differences among the etoposide-treated and G-CSF-only groups (Fig. 5a–b). To confirm that the changes in the plasma levels of KC, MIP-2, and LIX reflected similar changes in the BM, we quantified the IHC images of BM sections using the IHC profiler Plugin of ImageJ (G-CSF only, *n* = 7; cyclophosphamide + G-CSF, *n* = 7; etoposide + G-CSF, *n* = 7). The levels of KC, MIP-2, and LIX were all significantly increased in the BM sections from the etoposide-treated group, compared with those from the G-CSF-only and cyclophosphamide-treated groups (*p* < 0.001 and *p* < 0.001; *p* = 0.004 and *p* < 0.001; *p* < 0.001 and *p* < 0.001 after Bonferroni correction, respectively; Fig. 5c–e).

### Etoposide-induced chemo-mobilization is associated with increased MMP9 and decreased SDF-1 levels in the BM

**Fig. 6:**
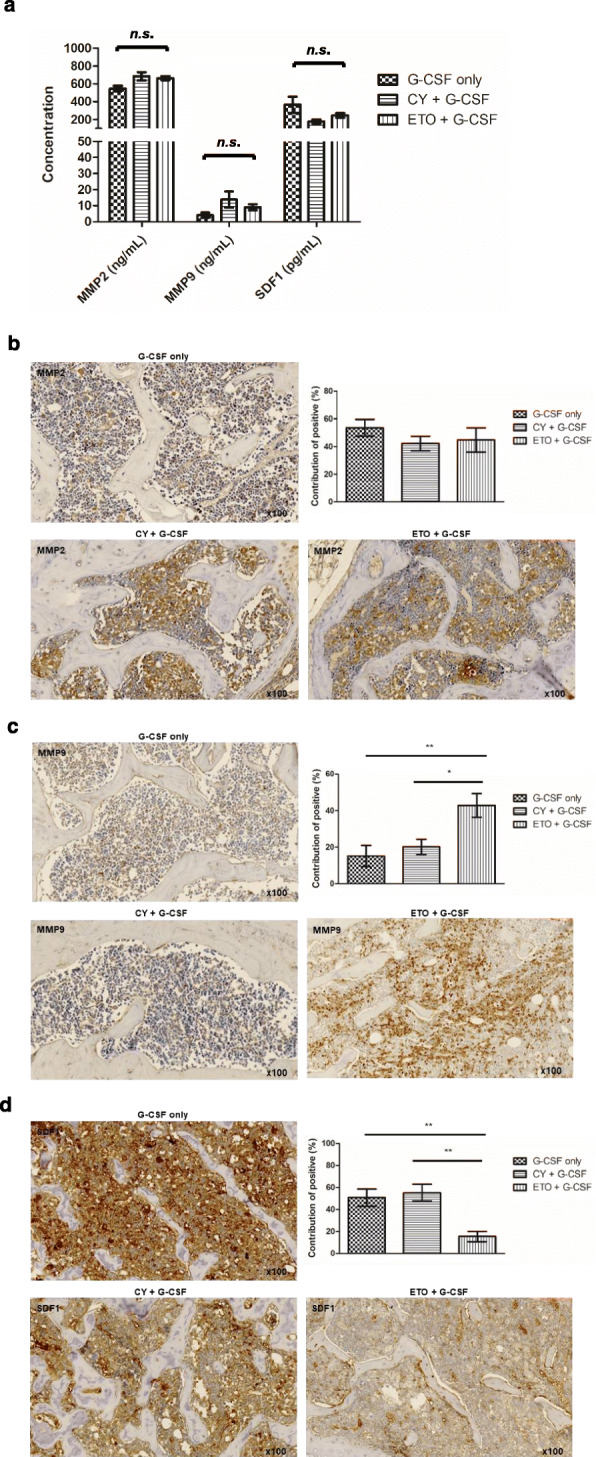
Matrix metalloprotease (MMP) 2, MMP9, and stromal cell-derived factor-1 (SDF-1) expression in the mouse model of peripheral blood hematopoietic stem cell mobilization. **a** MMP2, MMP9, and SDF-1 expression in the peripheral blood did not differ significantly among the groups (G-CSF only, *n* = 4; cyclophosphamide + G-CSF, *n* = 4; etoposide + G-CSF, *n* = 4). **b**, **c**, **d** In the bone marrow, the etoposide + G-CSF group showed a significant increase in MMP9 and decrease in SDF-1 expression, compared to the G-CSF only and cyclophosphamide + G-CSF groups (G-CSF only, *n* = 7; cyclophosphamide + G-CSF, *n* = 7; etoposide + G-CSF, *n* = 7). Note: ** *p* < 0.01 after Bonferroni correction; * *p* < 0.05 after Bonferroni correction. Note: Values are reported as the mean ± SEM. Abbreviations: G-CSF, granulocyte colony-stimulating factor; CY, cyclophosphamide; ETO, etoposide; n.s., not significant

The cytokine network was comprehensively analyzed to identify the potential mechanisms underlying etoposide-induced HSC mobilization. Cytokines exhibiting a significant (*p* < 0.05) increase in response to etoposide-induced chemo-mobilization, compared to that in response to G-CSF-only- or cyclophosphamide-induced chemo-mobilization, in mouse cytokine assays were analyzed by Ingenuity Pathway Analysis (Qiagen, Redwood City, CA, USA; Supplementary Material 4 and 5: Fig. S1 and S2). Network analysis showed that cytokines exhibiting increased levels in response to etoposide-induced chemo-mobilization were associated with the activation of matrix metalloproteinases (MMPs), which affect the CXCR4/SDF-1 axis, and are known to be involved in HSC mobilization [34, 35]. Therefore, the expression of MMPs related to HSC mobilization, i.e., MMP2 and MMP9, was assessed. In the PB, the expression of MMP2, MMP9, and SDF-1 did not differ significantly among groups (G-CSF-only, *n* = 4; cyclophosphamide chemo-mobilization, *n* = 4; etoposide chemo-mobilization, *n* = 4). However, in the BM, MMP9 expression was significantly increased and SDF-1 expression was significantly decreased in the etoposide-induced chemo-mobilization group, compared to that in the other groups (G-CSF-only, *n* = 7; cyclophosphamide + G-CSF, *n* = 7; etoposide + G-CSF, *n* = 7; Fig. 6).

## Discussion

Our retrospective analysis of clinical data showed that etoposide-induced chemo-mobilization results in the highest yield of CD34+ cells among all three groups analyzed, despite relatively modest changes in PB WBC counts. To our knowledge, this is the first analysis of clinical data pertaining to etoposide-induced chemo-mobilization. This study suggests the possibility of a different mechanism for chemo-mobilization by etoposide. Our in vitro experiments showed that etoposide significantly increased the secretion of IL-8 by hBMSCs, whereas cyclophosphamide did not. IL-8 is a part of the senescence-associated secretory phenotype; therefore, this finding might be associated with the specific influence of etoposide on hBMSC subcultures, which was not observed upon treatment with cyclophosphamide. This finding might provide a clue to explain the higher efficiency of etoposide at inducing chemo-mobilization compared to that of cyclophosphamide. Studies by Pelus et al. and Fukuda et al. support this hypothesis by showing that the CXCR2 ligand GRO-β rapidly mobilizes HSCs and enhances engraftment, although the underlying mechanism has not yet been elucidated [36]. Moreover, we had previously reported that CXCR2 (an IL-8 receptor) stimulation is crucial for maintaining the proliferation of human pluripotent stem cells (hPSCs) [34, 35]. Therefore, we hypothesized that IL-8 activates the proliferation of hHSCs in a manner similar to that of hPSCs, resulting in more efficient mobilization. To confirm this hypothesis, we performed an in vitro experiment to determine the effect of IL-8 on hHSCs in a simulated BM environment using conditioned medium from healthy hBMSCs; we observed an expansion of CD34+ and CD34+/CD45- cells. We also observed the concomitant significant enhancement in *CXCR2*, *mTOR*, and c-*MYC* expression in CD34+ cells following IL-8 stimulation.

Our finding that IL-8 stimulated CXCR2 and mTOR expression is consistent with the results of our studies on hPSCs [37], and with the observation that mTOR activates c-*MYC* [38]. With respect to the role of c-*MYC* in hematopoiesis, Wilson et al. reported that c-*MYC* controls the balance between stem cell self-renewal and differentiation, presumably by regulating the interaction between HSCs and their niche [39]. Laurenti et al. demonstrated that the loss of c-*MYC* alone resulted in the inability of HSCs to differentiate into progenitors; furthermore, the majority of the early and late progenitors stopped proliferating, resulting in HSC accumulation in the BM niche [40]. A study by Ehninger et al. showed that although HSCs express low levels of the c-MYC protein, its expression increases steadily during progenitor differentiation [41]. In a recent study, it was reported that IL-8 activates mTOR and increases endogenous c-MYC production, thereby inducing PDL1 expression in gastric cancer [42]. In the present study, IL-8 significantly increased not only the number of CD34+ cells but also that of CD34+/CD45- cells. The results of our previous studies that demonstrated the role of CXCR2 in supporting hPSC proliferation [34, 35], suggest that the activation of CXCR2 by IL-8 may have enhanced hHSC proliferation; however, further studies are necessary to confirm this hypothesis. Therefore, etoposide may induce IL-8 secretion from hBMSCs, which stimulates CXCR2 in HSCs, thereby activating mTOR and c-MYC and leading to HSCs proliferation and progenitor cell differentiation. To our knowledge, this is the first HSC mobilization study to report such a mechanism. Furthermore, this mechanism may also explain the excellent yield at PBSCC during chemo-mobilization induced using etoposide that was also associated with a modest change in WBC count in the PB.

In clinical practice, it is difficult to observe changes in the BM niche in patients undergoing PBSCC. Moreover, cytokine measurements in the PB do not always accurately reflect levels in the BM niche due to systemic confounding factors. To overcome these obstacles, we established standardized animal mobilization models that excluded such confounding factors. We were able to simulate the cyclophosphamide and etoposide mobilization patterns observed in clinical practice in two distinct mouse models. Using these models, we confirmed the significantly increased expression of IL-8 homologs and MMP9 and decreased expression of SDF-1 in the BM during etoposide-induced chemo-mobilization, compared to that during G-CSF only- and cyclophosphamide-induced chemo-mobilization. The levels of IL-8 homologs in the PB during G-CSF-induced mobilization were comparable to those during etoposide-induced chemo-mobilization. Watanabe et al. had previously reported that G-CSF increased the WBC counts and IL-8 levels during mobilization. Increased IL-8 levels were correlated with higher numbers of CD34+ cells in the PB [43]. G-CSF was associated with polymorphonuclear neutrophils, which leads to increased IL-8 levels, and potentially, mobilization [44–46]. Moschella et al. had reported that cyclophosphamide induced the transcriptional modulation of PB MNCs and IFN-1-related sterile inflammatory responses. In that study, the levels of IL-8, an IFN-1-induced proinflammatory mediator in the PB [47], also increased significantly. Thus, inflammatory response could be the reason underlying the increase in IL-8 levels in the PB after etoposide treatment; however, few studies have addressed this issue [48, 49]. Previous studies have reported that IL-8 is produced by phagocytes and mesenchymal cells exposed to inflammatory stimuli [50], and that etoposide affects the BMSCs [16, 51]. Therefore, increased IL-8 levels in the PB may be due to inflammatory responses as well as hBMSCs. Additionally, IL-8 may enhance MMP9 production [52], leading to SDF-1 degradation and subsequent mobilization [34, 53, 54]. The results of our animal study revealed that etoposide increases the expression of IL-8 homologs (KC, MIP-2, and LIX) and MMP9 and decreases SDF-1 expression in the BM, although the levels of these molecules in the PB were similar in all the groups. These findings suggested that the origin of IL-8 in the PB during etoposide-induced chemo-mobilization was predominantly the BM niche rather than systemic inflammation. Synthetically, etoposide stimulated hBMSCs to secrete IL-8, which activated CXCR2, mTOR, and c-MYC in the HSCs, resulting in their proliferation. Moreover, MMP9 levels increased and SDF-1 decreased in the BM niche, resulting in HSC mobilization.

The results of this study demonstrate that etoposide causes long-term hBMSC toxicity associated with cell-cycle arrest at the G0/G1 phase. Hare et al. had reported that exposure of hBMSCs to sub-lethal doses of etoposide resulted in an increased proportion of cells arrested at the G0/G1 phase [55]. Moreover, BMSCs could not activate non-homologous end-joining repair following etoposide-induced stress after successive passages [55]. Clinical data also suggest that the toxicity of etoposide in the BM niche is higher and lasts longer than that of cyclophosphamide [9, 10]. However, currently no definitive data are available regarding the adverse effects of etoposide on engraftment or survival. Studies on this topic need to be conducted in future.

Our present study has several limitations, the first of which is the absence of plerixafor + G-CSF, which can induce adequate of the HSCs mobilization with less toxicity. There are two reasons for proceeding without including plerixafor + G-CSF, i.e., (1) plerixafor is often difficult to use clinically in some countries at it is expensive, and (2) the mechanism of action of plerixafor is relatively well-known. For these reasons, we focused on comparing three chemo-mobilization methods (G-CSF only, cyclophosphamide + G-CSF, or etoposide + G-CSF) that have been used in clinical practice but whose mechanisms of action are unclear. Second, this study focused predominantly on cytokine or enzyme changes in the BM niche rather than systemic inflammation because previous studies have generally assessed the role of systemic inflammation in mobilization; moreover, we suspected that the effect of etoposide on the BM niche might be the main mechanism underlying HSC mobilization. To this end, we used healthy hBMSC-conditioned medium that reflects the environment of the normal BM niche, for culturing the CD34+ hHSCs. A study design investigating both the aspects of mobilization would be very complex. Nevertheless, to our knowledge, this is the first study on the mechanism of etoposide-induced chemo-mobilization that focuses on the BM niche. Additionally, this study describes the establishment of the first mouse model of etoposide-induced chemo-mobilization that reflects the conditions encountered in clinical practice.

## Conclusion

In conclusion, etoposide-induced chemo-mobilization is highly effective for harvesting HSCs from the PB. The mechanism of action of etoposide is associated with increased IL-8 secretion by hBMSCs, which induces the expansion of HSCs in a manner dependent on CXCR2, mTOR, and c-*MYC* activation as well as increase and decrease of MMP9 and SDF-1 levels, respectively in the BM niche. Finally, our results suggest that etoposide exposure should be minimized before and after PBSCT because of its long-term toxicity to hBMSCs. These findings emphasize the need for further studies to develop more efficient and safe chemo-mobilization strategies.

## Supplementary information

**Additional file 1.**

**Additional file 2.**

**Additional file 3.**

**Additional file 4.**

**Additional file 5.**

**Additional file 6.**

## Data Availability

All data generated or analyzed during this study are included in this published article and its supplementary information files.

## Abbreviations

Auto-PBSCT: Autologous peripheral blood stem cell transplantation
BM: Bone marrow
CFU: Colony-forming unit
Cmax: Maximum reported serum concentration
G-CSF: Granulocyte colony-stimulating factor
hBMSCs: Human bone marrow stromal cells
hHSCs: Human hematopoietic stem cells
HPC: Hematopoietic progenitor cells
hPSC: Human pluripotent stem cell
IHC: Immunohistochemical
KC: Keratinocyte-derived cytokine
LIX: Lipopolysaccharide-inducible CXC chemokine
MIP-2: Macrophage inflammatory protein 2
MMP: Matrix metalloproteinase
MMP2: Matrix metalloproteinase-2
MMP9: Matrix metalloprotease-9
MNC: Mononuclear cells
NHL: Non-Hodgkin’s lymphoma
PB: Peripheral blood
PBSCC: Peripheral blood stem cell collection
SDF-1: Stromal cell-derived factor-1
WBC: White blood cell
