# Supplementary material for: Etoposide-mediated interleukin-8 secretion from bone marrow stromal cells induces hematopoietic stem cell mobilization
Source: BMC Cancer. 2020 Jul 2;20:619. doi: 10.1186/s12885-020-07102-x (PMC7330970; doi:10.1186/s12885-020-07102-x)
Supplement: Supplementary file 1 — Additional file 1. [file 12885_2020_7102_MOESM1_ESM.docx]

**Table S1. Efficacy of etoposide-mediated mobilization protocols according to dose**

| **Trial (First investigator)** | **Year** | **Disease** | **Total dose of etoposide (*n*)** | **Control group (*n*)** | **≥ 2×10^6^ CD34^+^ cell (*n* (%))** | **≥ 5×10^6^ CD34^+^ cell (*n* (%))** |
| --- | --- | --- | --- | --- | --- | --- |
| Hyun *et al.* [10] | 2014 | NHL | 1,500 mg/m^2^  (total 29) | Cyclophosphamide 4.0 g/m^2^ (total 31) | 29 (100%) vs. 25 (81%) | 25 (86%) vs. 14 (45%) |
| Wood *et al.* [11] | 2013 | Lymphoma  (NHL or HL) | 750 mg/m^2^ with 10 μg/kg/day G-CSF  (total 159) | NA | 150 (94%) | 90 (57%) |
| Wood *et al.* [12] | 2011 | MM | 750 mg/m^2^ with 10 μg/kg/day G-CSF  (total 152) | NA | 152 (100%) | 150 (99%) |
| Milone *et al.* [9] | 2007 | Lymphoma  (NHL or HL) | 600 mg/m^2^ with 16 μg/kg/day G-CSF  (total 41) | Cyclophosphamide 4.0 g/m^2^ with 16 μg/kg/day G-CSF (total 37) | 35 (85.3%) vs. 26 (70.2%) | NA |
| Park *et al.* [13] | 2019 | MM | 375 mg/m2 with 10 μg/kg/day G-CSF  (E375, total 32) | 4 days of 10 μg/kg/day G-CSF alone (total 57)  Cyclophosphamide 3.5 g/m^2^ with 10 μg/kg/day G-CSF (total 48)  Etoposide 750 mg/m^2^ with 10 μg/kg/day G-CSF (E750, total 31) | 29 (90.3%) vs.  52 (91.2%)  42 (87.5%)  31 (100%) | 26 (83.9%) vs.  17 (29.8%)  33 (68.8%)  23 (71.9%) |

**Abbreviations:** NHL, non-Hodgkin lymphoma; ANC, absolute neutrophil counts; G-CSF, granulocyte colony-stimulating factor; HL, Hodgkin lymphoma; NA, not applicable; RBC, red blood cell; MM, multiple myeloma.
