# Supplementary material for: Etoposide-mediated interleukin-8 secretion from bone marrow stromal cells induces hematopoietic stem cell mobilization
Source: BMC Cancer. 2020 Jul 2;20:619. doi: 10.1186/s12885-020-07102-x (PMC7330970; doi:10.1186/s12885-020-07102-x)
Supplement: Supplementary file 5 — Additional file 5. [file 12885_2020_7102_MOESM5_ESM.pptx]

## Slide 1
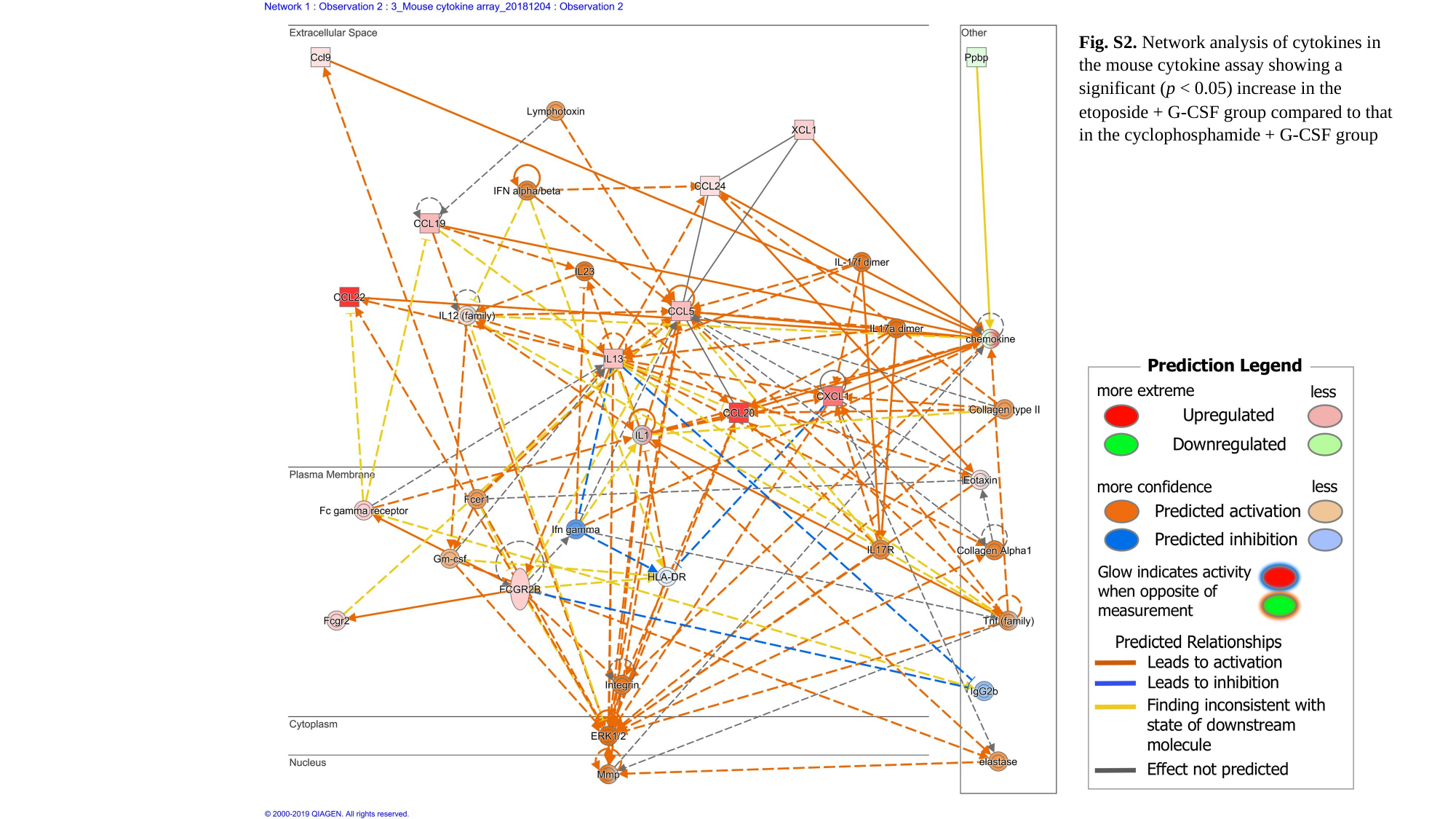

Fig. S2. Network analysis of cytokines in the mouse cytokine assay showing a significant (p < 0.05) increase in the etoposide + G-CSF group compared to that in the cyclophosphamide + G-CSF group
