# Supplementary material for: Etoposide-mediated interleukin-8 secretion from bone marrow stromal cells induces hematopoietic stem cell mobilization
Source: BMC Cancer. 2020 Jul 2;20:619. doi: 10.1186/s12885-020-07102-x (PMC7330970; doi:10.1186/s12885-020-07102-x)
Supplement: Supplementary file 6 — Additional file 6. [file 12885_2020_7102_MOESM6_ESM.pptx]

## Slide 1
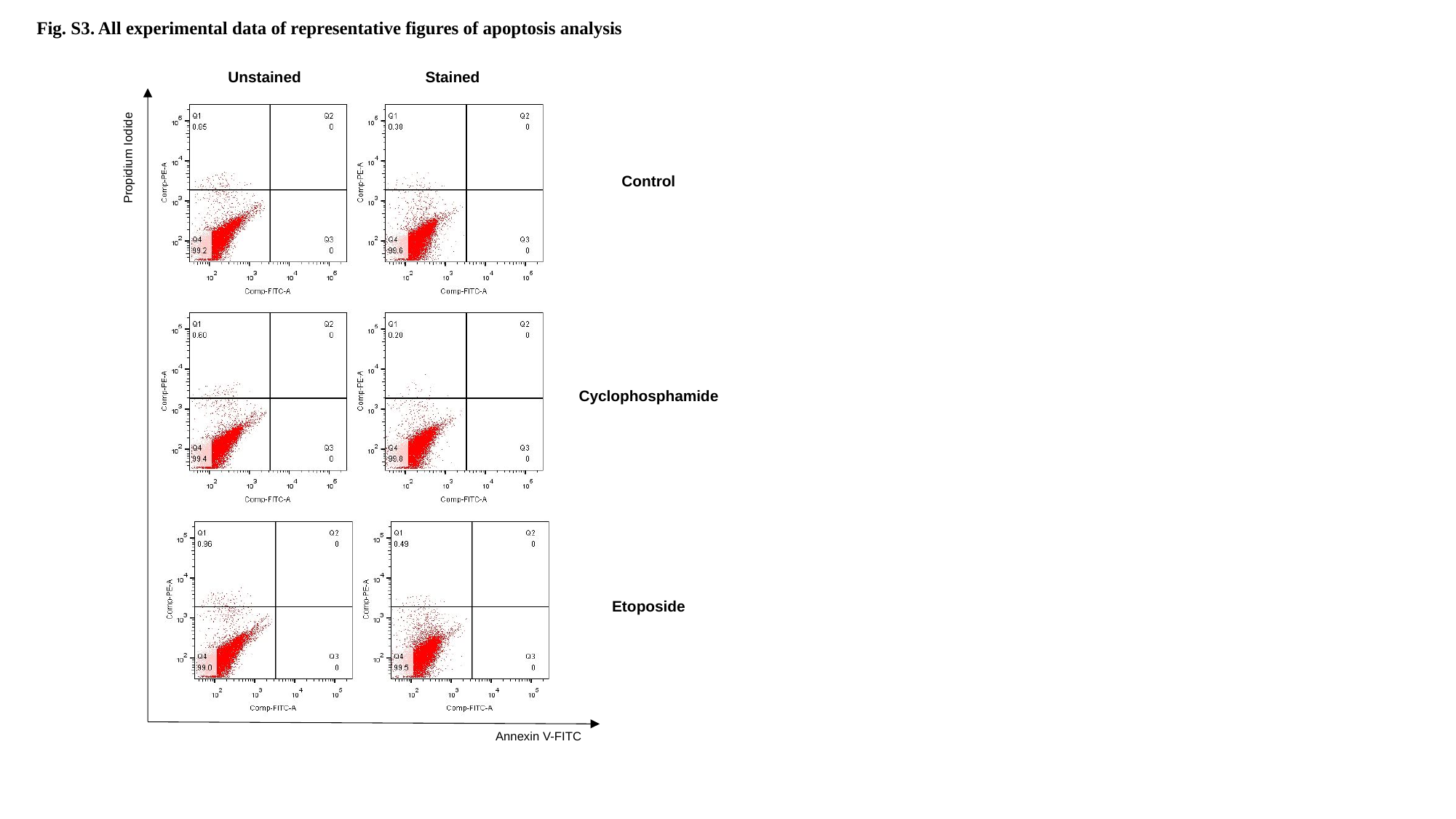

Fig. S3. All experimental data of representative figures of apoptosis analysis
Unstained
Stained
Propidium Iodide
Control
Cyclophosphamide
Etoposide
Annexin V-FITC
